# Repositioning of Omarigliptin as a once-weekly intranasal Anti-parkinsonian Agent

**DOI:** 10.1038/s41598-018-27395-0

**Published:** 2018-06-12

**Authors:** Bassam M. Ayoub, Shereen Mowaka, Marwa M. Safar, Nermeen Ashoush, Mona G. Arafa, Haidy E. Michel, Mariam M. Tadros, Mohamed M. Elmazar, Shaker A. Mousa

**Affiliations:** 1 Pharmaceutical Chemistry Department, Faculty of Pharmacy, The British University in Egypt, El-Sherouk city, Cairo Egypt; 2 The Center for Drug Research and Development (CDRD), Faculty of Pharmacy, The British University in Egypt, El-Sherouk city, Cairo Egypt; 3 Analytical Chemistry Department, Faculty of Pharmacy, Helwan University, Ein Helwan, Cairo Egypt; 4 Pharmacology & Biochemistry Department, Faculty of Pharmacy, The British University in Egypt, El-Sherouk city, Cairo Egypt; 5 Pharmacology & Toxicology Department, Faculty of Pharmacy, Cairo University, Kasr El-Aini st., Cairo, Egypt; 6 Clinical Pharmacy and Pharmacy Practice Department, Faculty of Pharmacy, The British University in Egypt, El-Sherouk city, Cairo Egypt; 7 Pharmaceutics Department, Faculty of Pharmacy, The British University in Egypt, El-Sherouk city, Cairo Egypt; 8 Chemotheraputic Unit, Mansoura University Hospitals, Mansoura, 35516 Egypt; 9 Pharmacology & Toxicology Department, Faculty of Pharmacy, Ain Shams University, El-Abaseya, Cairo Egypt; 10 Analytical Chemistry Department, Faculty of Pharmacy, Ain Shams University, El-Abaseya, Cairo Egypt; 11 The Pharmaceutical Research Institute, Albany College of Pharmacy and Health Sciences, Rensselaer, NY United States

## Abstract

Drug repositioning is a revolution breakthrough of drug discovery that presents outstanding privilege with already safer agents by scanning the existing candidates as therapeutic switching or repurposing for marketed drugs. Sitagliptin, vildagliptin, saxagliptin & linagliptin showed antioxidant and neurorestorative effects in previous studies linked to DPP-4 inhibition. Literature showed that gliptins did not cross the blood brain barrier (BBB) while omarigliptin was the first gliptin that crossed it successfully in the present work. LC-MS/MS determination of once-weekly anti-diabetic DPP-4 inhibitors; omarigliptin & trelagliptin in plasma and brain tissue was employed after 2 h of oral administration to rats. The brain/plasma concentration ratio was used to deduce the penetration power through the BBB. Results showed that only omarigliptin crossed the BBB due to its low molecular weight & lipophilic properties suggesting its repositioning as antiparkinsonian agent. The results of BBB crossing will be of interest for researchers interested in Parkinson’s disease. A novel intranasal formulation was developed using sodium lauryl sulphate surfactant to solubilize the lipophilic omarigliptin with penetration enhancing & antimicrobial properties. Intranasal administration showed enhanced brain/plasma ratio by 3.3 folds compared to the oral group accompanied with 2.6 folds increase in brain glucagon-like peptide-1 concentration compared to the control group.

## Introduction

Parkinson’s disease (PD) is a neurodegenerative disease^1^. Glucagon-like peptide-1 (GLP-1) was reported as a potential candidate in modifying neurodegenerative diseases as a promising antiparkinsonian effect of dipeptidyl peptidase (DPP)-4 inhibitors (Gliptins) by exerting a neuroprotective effect in PD animal models^2,3^. Sitagliptin^4–6^, vildagliptin^7^, saxagliptin^8^ & linagliptin^9^ showed anti-oxidant, anti-apoptotic and neuro-restorative mechanisms in previous studies linked to DPP-4 inhibition^10^. Moreover, a recent study suggested repositioning of teneligliptin to brain disorders^11^. Interestingly, omarigliptin (OG) & trelagliptin (TG) in this study were considered for the first time to test their ability to cross the blood brain barrier (BBB) suggesting OG repositioning to brain disorders based on its BBB crossing, its polypharmacology and potential increasing of GLP-1 concentration in the brain.

Drug repositioning is a hot research topic as an alternative to underperforming hypothesis-driven molecular target based drug discovery efforts^12–15^. De novo drug discovery is a traditional approach, which is costly and time-consuming process. Thus, drug repositioning was an alternative approach as therapeutic switching or drug repurposing for already marketed drug with less time consuming and less costly^16^. It has proved to be a preferred strategy for accelerated drug discovery as a relatively inexpensive pathway that carries minimal risk due to availability of previous pharmacological, safety and toxicology data^17^ with many successful suggested studies in the literature^18–33^.

**Figure 1:**
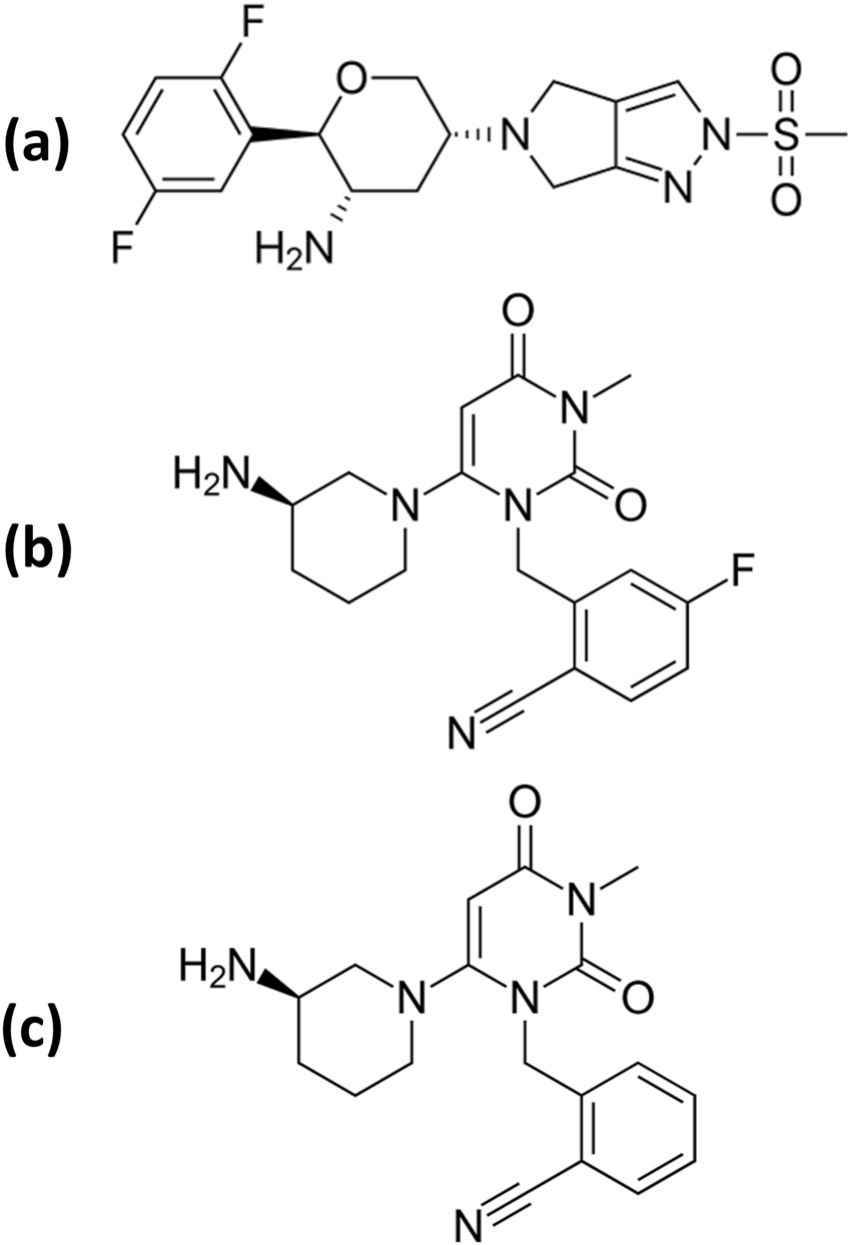
Chemical structures of omarigliptin (**a**) trelagliptin (**b**) and the internal standard, alogliptin (**c**).

OG (Fig. 1a) and TG (Fig. 1b) are new once weekly anti-diabetic drugs. Despite the fact that the initial therapy of diabetes usually be with metformin, thereafter treatment should consider different second line options. These include DPP-4 inhibitors, of which OG and TG are once weekly versions^34,35^. In contrast to the once-daily DPP-4 inhibitors, once-weekly administration can improve patients’ adherence^36–43^.

In the present work, sensitive and specific LC-MS/MS methods were developed and validated for estimation of OG & TG in rats’ plasma and brain tissue to show their interaction with the BBB to check for the possibility of their repositioning as antiparkinsonian agents. As per FDA guidelines^44^, a detailed validation of the LC-MS/MS methods was carried out. The proposed repositioning study of OG, after the proof of crossing BBB, will be of interest for pharmaceutical industry & researchers working in the area of PD treatment with the major advantages of repositioning that include safety, saving time & money. Preliminary investigations confirmed that alogliptin is a suitable internal standard (IS) with similar physical and chemical properties while performing the simple sample extraction procedures^45–47^ as shown in its structure presented in Fig. 1c.

Determination of drugs in animal brain tissue is common in the literature^48–58^ to check their crossing of BBB. Various extraction techniques were employed for extraction of drugs either from brain homogenate alone^50,51^ or from both animal plasma & brain extract^50–56^ including direct precipitation^48–51^; liquid-liquid extraction^52–56^, solid phase extraction^57^ & QuEChERS based approach^58^. Moreover, direct precipitation was also used for simultaneous extraction of eight neurotransmitters from brain tissue^59^. Results showed that OG crossed the blood brain barrier (BBB) suggesting repositioning as antiparkinsonian agent. Moreover, a novel intranasal formulation was developed using sodium lauryl sulphate surfactant to solubilize the lipophilic omarigliptin with penetration enhancing & antimicrobial properties. Intranasal administration to rats showed enhanced brain/plasma ratio by 3.3 folds than the oral group accompanied with 2.6 folds increase in brain glucagon-like peptide-1 (GLP-1) concentration than the control group. Furthermore, the developed method used with rat plasma was extended to human plasma and applied for bioassay of samples from twelve human volunteers. Because of change in the species, it necessitated a partial validation study as the results of human QC samples showed (10–13%) lower recoveries than rat samples, which might be attributed to higher binding affinity of the drugs to human plasma proteins due to species difference^60^.

## Methods

### Chemicals and reagents

Human plasma, OG & TG (99.0%), MARIZEV (25 mg) & ZAFATEK (100 mg) tablets were kindly donated by the Center for Drug Research and Development (CDRD, BUE) from a previous project fund. HPLC grade acetonitrile, HPLC water & formic acid were purchased from Sigma Aldrich (USA). Sandwich ELISA kit (CUSABIO, CSB-E08117r) was used for GLP-1 determination in rats’ brain tissue samples. Potassium dihydrogen phosphate was purchased from VWR Chemicals (Pool, England). The following surfactants were used as 2.5% (*w/v*) aqueous solutions: Sodium Lauryl Sulphate (SLS) and Tween-80 from (El-Nasr Pharmaceutical Chemicals Co., Cairo, Egypt).

### LC-MS/MS conditions

The same LC-MS/MS instrument & chromatographic conditions described by the same authors (Bassam Ayoub & Shereen Mowaka) for TG & IS (alogliptin) assay^61^ were adopted and extended to include OG in the current work. Furthermore, collision energy of 30 eV and cone voltage of 30 V was found to be suitable for MRM of OG using the same other reported mass detection parameters^61^. “WATERS UPLC system (USA), TQ detector supplemented with electrospray ionization source (USA) and Agilent SB-C_18_ column with dimensions (1.8 µm) 50 × 2.1 mm was used. Mass Lynx software version 4.1 was used. A mixture of acetonitrile - formic acid 0.1% (80:20, *v/v*) was used as the mobile phase, filtered via a filter membrane with 0.2 µm pore size and it was degassed for 25 min. Injection volume of 7.5 µL and flow rate of 0.3 mL/min were applied successfully”^61^. A run time of 2 min was used keeping the column temperature at 25 °C. Multiple reaction monitoring (MRM) of the transition pairs of m/z 399.1 to 153.0 for OG, m/z 358.2 to 134.1 for TG and m/z 340.2 to 116.1 for IS in the positive mode utilizing Electro Spray Ionization (ESI) was implemented^62^. “The following parameters were applied: turbo ions spray at 400 °C, capillary temperature at 275 °C, sheath and auxiliary gas at 15 and 2 psi, respectively, ion spray voltage of 3800 V, capillary voltage of 4 KV, capillary offset of 35 and de-solvating line temperature at 400 °C”^62^.

### Calibrators and QC samples

Stock solutions of OG & TG (1 mg/mL) were prepared separately in methanol and serially diluted with methanol to prepare working solutions of OG & TG (0.5, 1.2, 1.5, 7.0, 14.0, 15.0, 18.0, 25.0 & 28.0 µg/mL) and stored at 4 °C. Ten microliters of the prepared working solution was spiked with 90 µL blank plasma or blank brain homogenate (10%) to make the corresponding standard or QC samples. The final concentrations for calibrators were 50 (LLOQ), 150, 700, 1400, 1800 and 2800 ng/mL, and for QC samples were 120 (LQC), 1500 (MQC) and 2500 ng/mL (HQC). Protein precipitation with acetonitrile was used for the processing of the calibrators & QC samples with full details under the sample preparation section. Calibration curves were obtained by plotting Peak Area Ratios (PAR) of each drug to IS, against the corresponding concentrations (C) of the drug.

### Sample preparation

A reported direct extraction method was used^63,64^ for both OG and TG in the presence of the IS after simple modification so it can be applied for both rats’ plasma and brain homogenate samples. An aliquot of 100 μL of plasma sample (or brain homogenate 10%) was spiked with 10 μL IS (10 μg/mL), precipitated with 400 μL acetonitrile, vortexed for 2 min, centrifuged at 12.000 rpm for 10 min, then 300 μL of the clear supernatant layer was diluted with 300 μL water, and stored at −80 °C waiting for analysis^63,64^.

### Bioanalytical validation

Validation was accomplished using US-FDA guidelines by investigating different QC levels (n = 3). Six calibrators were constructed to satisfy linearity. The final brain samples’ concentrations were calculated as ng/g brain tissue after considering the dilution factor of 10. Accuracy and precision were evaluated by analysis different QC samples three times a day (n = 3) & three times on different days (n = 3). The relative error (RE) and percent relative standard deviations (% RSD) were calculated. Selectivity of the method was checked by comparing the blank samples with the zero samples and *in vivo* samples’ chromatograms to ensure that there is no suppressing interference. Carry over effect was evaluated by injecting high concentration samples after the blank. Matrix effect was estimated based on the ratio of AUP of post-extracted QC samples & their corresponding pure solutions while the extraction recovery was estimated by the comparison of the AUP of extracted QC samples against their post-extracted samples. Moreover, stability of QC samples in the auto-sampler and short-term stability (room temp., 3 h), Freez-thaw cycles (n = 3) and long-term stability (−80 °C, 2 weeks) were tested.

### *In vivo* BBB crossing test and determination of brain GLP-1 concentration

Twenty-four rats (200 grams ± 25) were used in this study. They were randomly allocated into four groups (n = 6); the first received OG (5 mg/kg, p.o), the second received TG (20 mg/kg, p.o), the third received OG (5 mg/kg, intra-nasal) while the fourth group was kept as control. Intranasal administration was delivered as previously described by Yang *et al*.^65^. Briefly, rats were anesthetized with pentobarbital sodium (30 mg/kg, i.p.) and kept in a supine position with neck extended. A volume of 50 μl containing OG was applied to each naris using a 10 μl fine pipette tip. The dose was calculated for rats according to FDA guidelines for human-rodent dose conversions^66^ & the safety profile for OG in humans^67^. The proposed study considered the LC-MS/MS quantitative determination of OG & TG in plasma and brain tissue after oral administration to rats (n = 6 for each drug) and evaluated the significant difference for the intra-nasal route of administration for OG (n = 6) against the oral route by calculating the brain/plasma ratio for each route. Moreover, a control group (n = 6) was considered while comparing the GLP-1 brain concentration after the intranasal administration.

The main aim of the study was to check the crossing ability of the mentioned drugs for the BBB and to confirm that the developed LC-MS/MS method is applicable for the bioassay of the drugs in the actual biological samples (plasma & brain tissue) after 2 hours. The design of the study is one treatment, one period, single dose study. All procedures employed in this study were reviewed and approved by the ethics committee of the British University in Egypt. It is worthy to mention that tween 80 surfactant (2.5%, *w/v*) was required for OG suspension in saline (p.o) due to its hydrophobic properties while TG was very soluble. Sodium lauryl sulphate (2.5%, *w/v*) was used for the intra-nasal solution to dissolve OG enhancing its penetration and as antimicrobial agent.

After 2 hours of drugs administration, 0.3 mL blood samples were collected into heparinized tubes via rats’ tail vein (except the control group). The separated plasma (>100 µL) was pipetted to clean tubes and stored at −80 °C until analysis. All twenty four rats were then sacrificed and the whole brain of each animal was separated, washed in saline, homogenized (10%, *w/v* in saline) and kept frozen at −80 °C until LC-MS/MS analysis (groups I-III) & GLP-1 ELISA kit analysis (groups III & IV). The brain homogenate of groups III & IV was centrifuged at 3000 rpm for 3 minutes^68^ then the supernatant was used for determination of GLP-1 concentration using Sandwich ELISA kit (CUSABIO, CSB-E08117r) according to a reported method^69^. The dilution factor of ten was considered for all brain tissue calculations (LC-MS/MS & ELISA).

### Statistical analysis

Statistical analysis was performed using a software program (GraphPad Prism, version 5.01, Inc., 2007, San Diego, CA, USA). GLP-1 results were expressed as the mean ± SEM and analyzed using two-tailed Student’s t-test test. Probability values of less than 0.05 were considered statistically significant.

### Ethics statement

All experiments and methods were performed in accordance with relevant guidelines and regulations. All experimental protocols were reviewed, approved, signed & stamped by a named institutional ethical committee (The British University in Egypt). Furthermore, after partial validation of the described above method, it was extended to human subjects’ application. The ethics committee of the British University in Egypt approved the experimental protocols and informed consents. Moreover, the protocol was registered in clinicaltrials.gov (ID: NCT03362398).

## Results

All the calibrators & validation results are shown in (Table 1) in accordance with FDA bioanalytical guidelines^44^. Concentration of OG & TG in rats’ plasma (after 2 h, p.o) were found to be 2688.79 & 1754.79 ng/mL, respectively, calculated from the bio-analysis regression equations mentioned in (Table 1), which is in agreement with previously developed OG & TG pharmacokinetic studies in rats^63,64^. Only OG crossed the BBB after the oral administration showing concentration of 621.75 ng/g in brain tissue after considering the dilution factor. The brain/plasma concentration ratio of 0.23 (621.75/2688.79) was used to deduce the penetration power through the BBB. Results showed that only OG crossed the BBB efficiently suggesting its possible repositioning as antiparkinsonian agent that will be of interest for researchers interested in Parkinson’s disease. Intra-nasal administration of OG showed significant higher brain/plasma concentration ratio of 0.76 (609.83 ± 103.16 ng/g brain tissue/802.35 ± 76.85 ng/mL plasma expressed as mean ± S.E.M) enhancing the ratio by 3.3 folds compared to the oral route. Mean GLP-1 brain tissue concentration (±S.E.M), for the intranasal group, was found to be 69.32 ± 7.18 pg/g tissue against 26.87 ± 1.59 pg/g for the control group with a significant increase of 2.6 folds compared to the control group (*p* < 0.001). The dilution factor of ten was considered for all brain tissue calculations (LC-MS/MS & ELISA).

**Table 1:** Results obtained for the described LC-MS/MS method for determination of OG and TG in rats’ plasma & brain tissue.

Item	Rats’ plasma		Rats’ brain tissue	
	OG	TG	OG	TG
Regression Eq.	Y = 0.0005 × −0.0124	Y = 0.0007 × −0.0284	Y = 0.0005 + 0.0161	Y = 0.0007 × −0.0081
Correlation Co.	0.9994	0.9992	0.9980	0.9983
*LLOQ (ng/mL)	50	50	50	50
*LQC (ng/mL)	120	120	120	120
*MQC (ng/mL)	1500	1500	1500	1500
*HQC (ng/mL)	2500	2500	2500	2500
Accuracy (*RE)	−14.08 to +15.26	−10.82 to +19.07	−12.24 to +8.53	−13.1 to +7.98
Precision (*% RSD)	14.22–14.64	13.07–13.73	8.62–12.53	7.7–14.04
*ME	85.99–88.74	90.63–98.55	89.44–99.66	91.77–100.92
ME for IS	84.54–89.16		92.49–94.61	
Recovery %	87.99–91.03	77.36–92.52	55.29–89.52	58.90–91.55
Recovery % for IS	73.83–87.64		55.01–85.02	
Stability studies	R% (84.33–95.09)	R% (85.92–97.42)	R% (70.35–89.96)	R% (86.90–91.55)

*Where LLOQ is the lower limit of quantification, LQC is the low quality control sample, HQC is the high quality control sample, RE is the accuracy relative error, % RSD is the percent relative standard deviation, ME is the matrix effect & IS (the internal standard).

## Discussion

The doses for the underlying repositioning investigation were selected based on previously reported pharmacokinetic studies^63,64^ and calculated according to FDA human-rats dose conversions’ calculations^66^. A dose of 5 mg/kg OG for rats will be multiplied by 60 (average human kg) and then divided over 6.2 based on FDA body surface area conversions^66^ resulting in a human dose nearly equals to 50 mg with a reported well tolerated and safety profile^67^.

**Figure 2:**
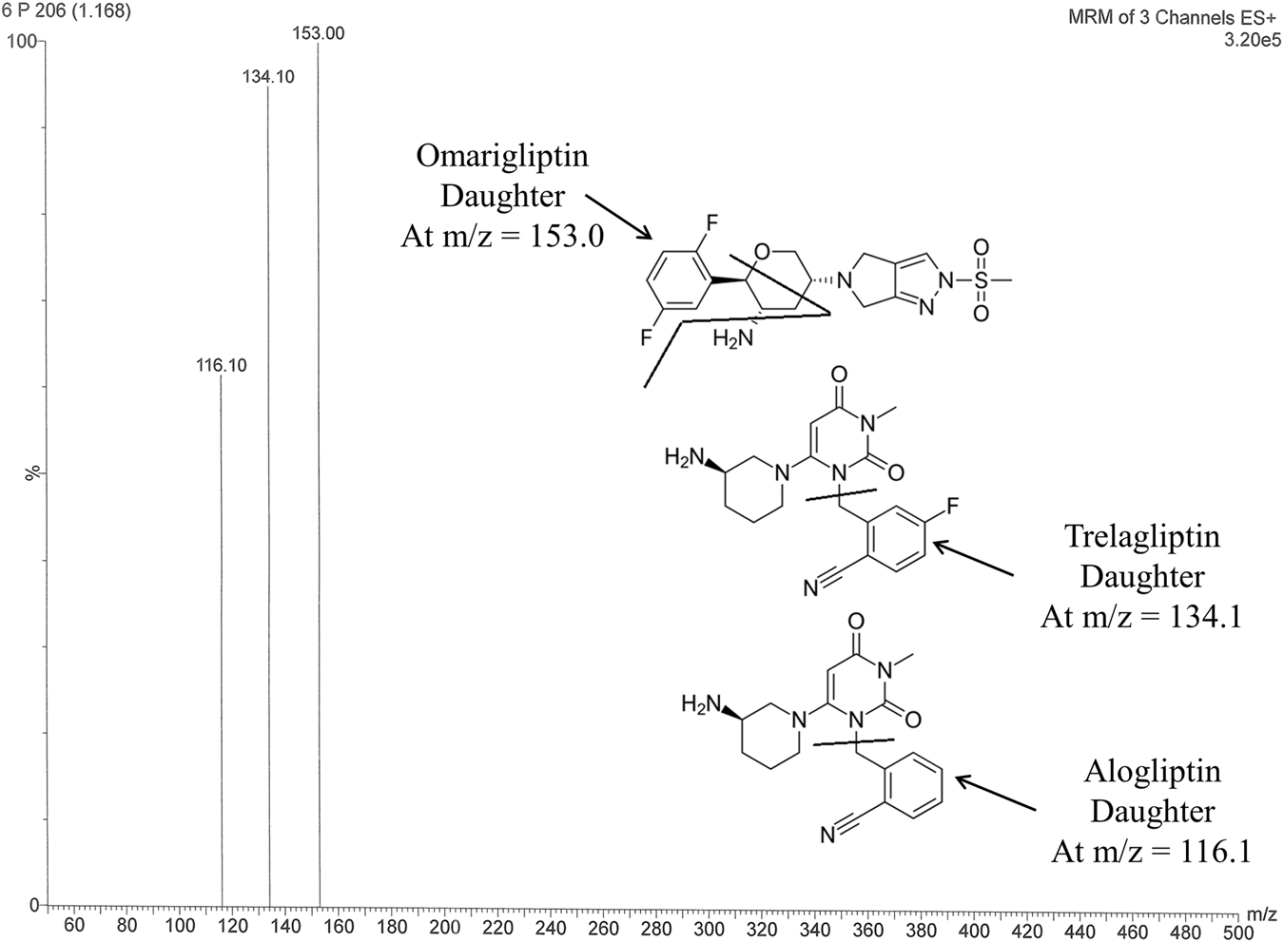
Daughter ions mass spectra in positive ESI ion detection mode with the proposed fragments showing m/z at 153.0, 134.1 & 116.1 for omarigliptin, trelagliptin & alogliptin, respectively.

In the present bio-analysis work, OG determination was not studied in the presence of other drugs or metabolites, as it is not a victim of drug-drug interactions or metabolism^70^. Although many direct precipitation^63,64,70^ and liquid-liquid^35,40,42,71,72^ extraction procedures were described in literature for OG and TG bioanalytical assays, simple direct precipitation with acetonitrile^63,64^ was selected by the authors and applied successfully for rats’ plasma and extended to the brain homogenate experiments. Figure 2 shows the suggested fragmentation pattern & daughters for the drugs displaying m/z 153.0 for OG, m/z 134.1 for TG and 116.1 for IS in the positive mode ESI. The same instrument, column, mobile phase & all the mass detection parameters were adopted from previously reported methods by the same authors^61,62^. Modification of the extraction procedure by applying 400 µL acetonitrile and further dilution with 300 µL water enabled similar retention times for both the plasma and brain homogenate extracts that was useful to evaluate accurately which drug crossed the BBB. Literature showed that sitagliptin and linagliptin did not cross the BBB^73,74^ so working on BBB crossing ability is an interesting point for gliptins especially after the reported anti-parkinsonian activity of the previously developed gliptins^4–9^.

**Figure 3:**
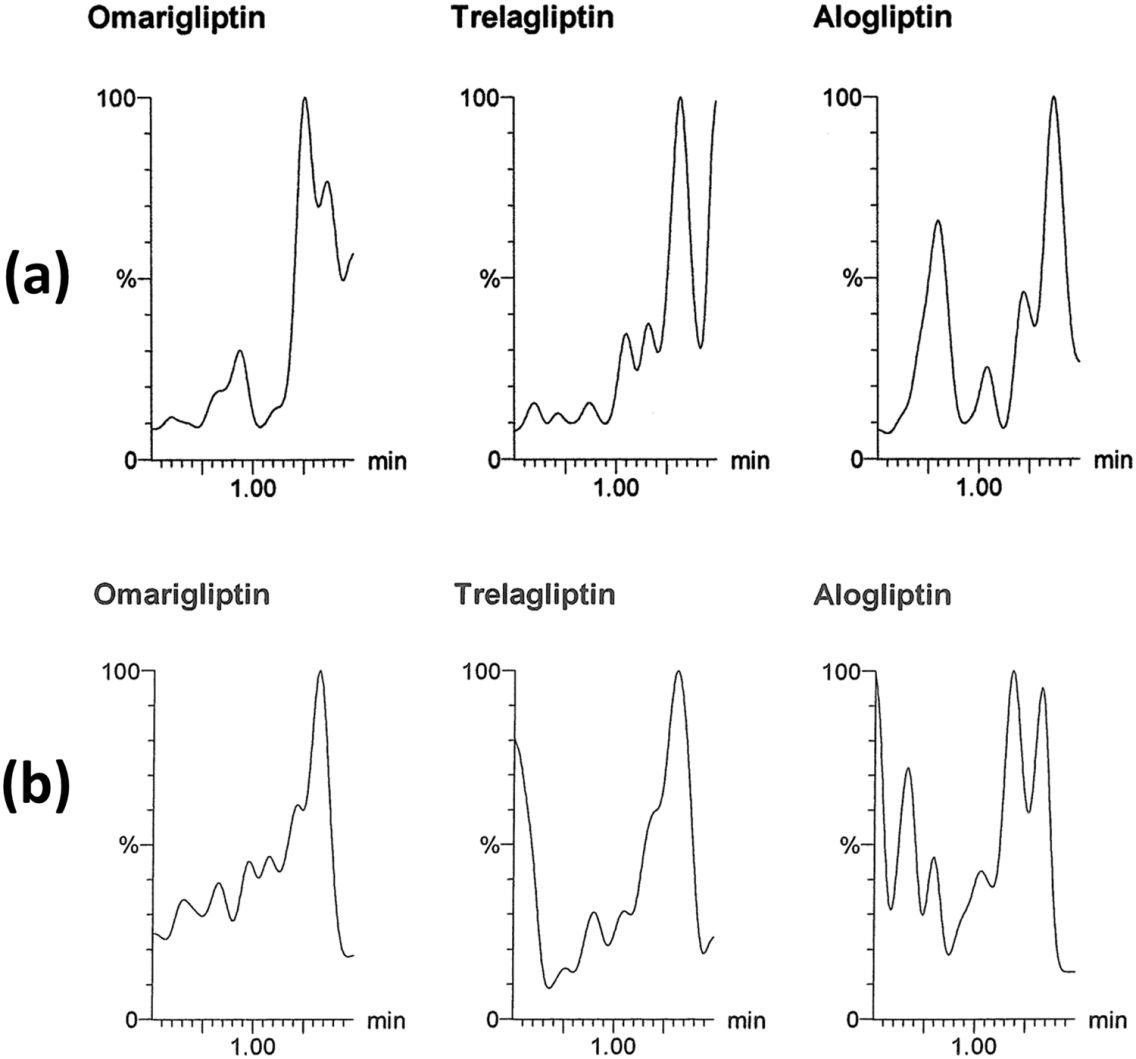
Blank plasma (**a**) and blank brain homogenate (**b**) samples using LC-MS/MS.

**Figure 4:**
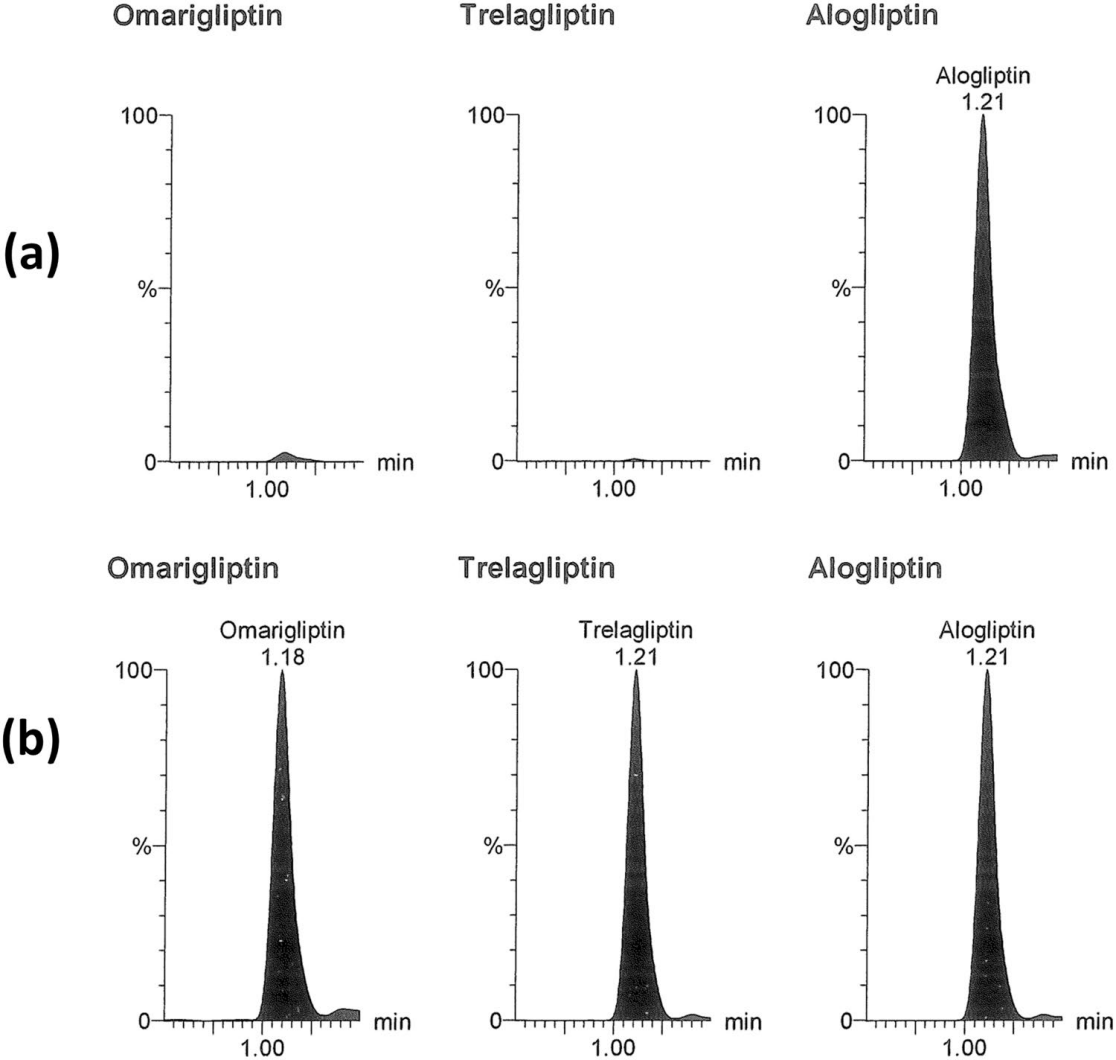
Multiple reaction monitoring (MRM) chromatogram of omarigliptin (m/z = 399.1 to 153.0), trelagliptin (m/z = 358.2 to 134.1) and alogliptin (internal standard, m/z = 340.2 to 116.1): (**a**) zero plasma spiked with internal standard; (**b**) plasma sample spiked with the three drugs at their lower limit of quantitation (LLOQ).

**Figure 5:**
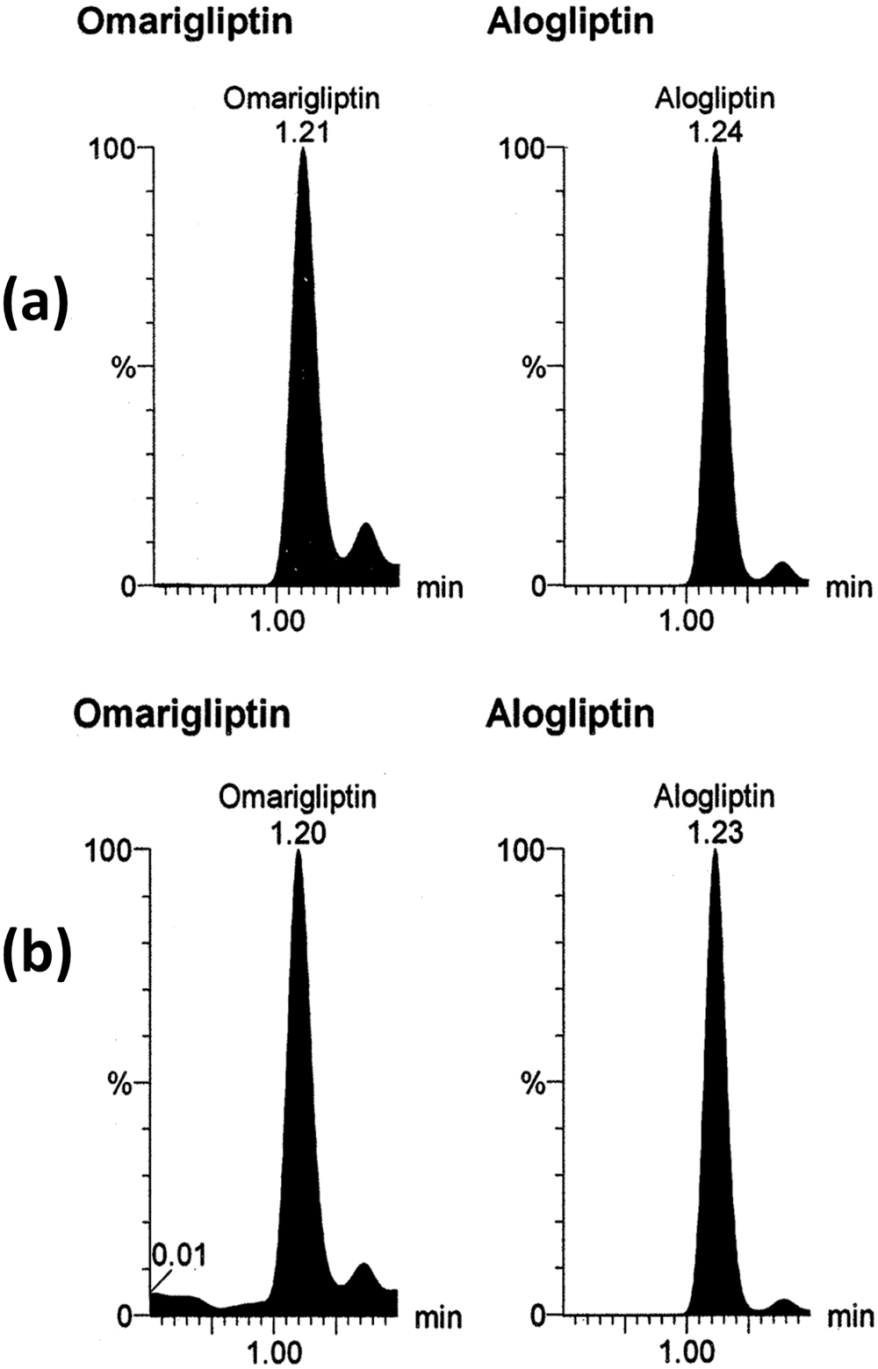
(**a**) MRM chromatogram of *in vivo* rat plasma sample obtained 2 hours after oral administration of omarigliptin (5 mg/Kg). (**b**) MRM chromatogram of *in vivo* rat brain homogenate sample obtained 2 hours after oral administration of omarigliptin (5 mg/Kg).

**Figure 6:**
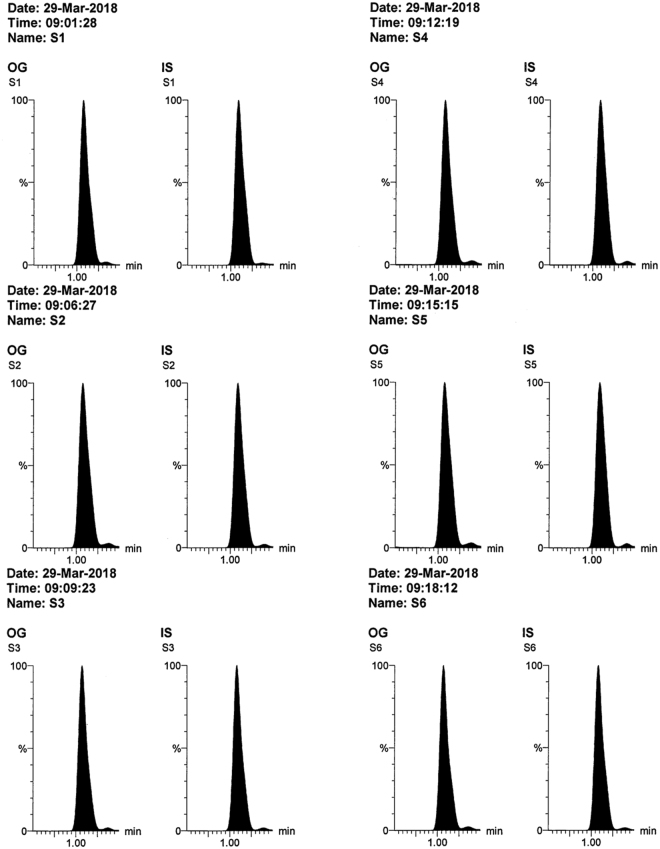
MRM chromatograms of *in vivo* rats’ brain homogenate 10% samples (n = 6) obtained 2 hours after intra-nasal administration of omarigliptin (5 mg/Kg) showing concentration of 609.83 ng/g tissue ± 103.16 expressed as mean ± S.E.M (after considering the dilution factor of 10).

**Figure 7:**
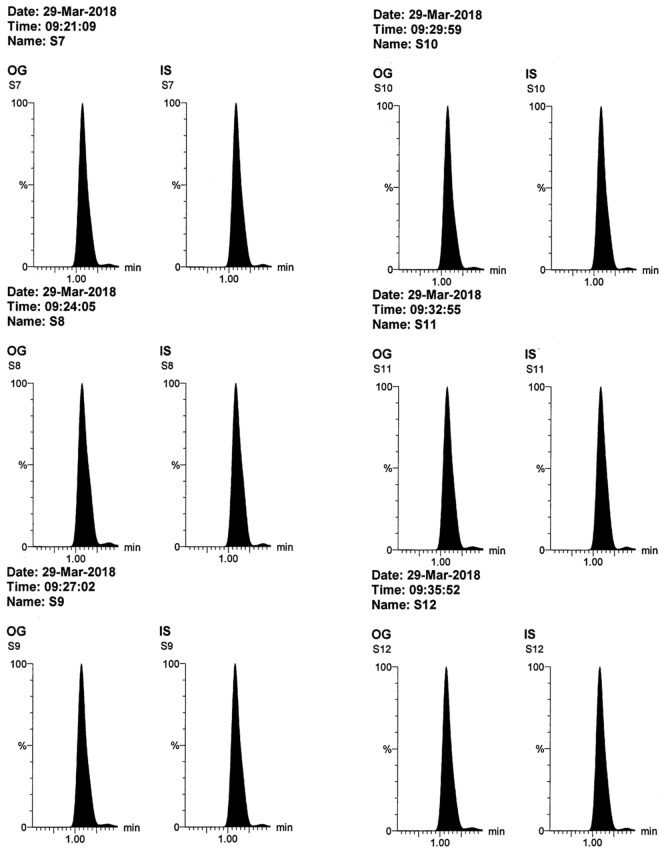
MRM chromatograms of *in vivo* rats’ plasma samples (n = 6) obtained 2 hours after intra-nasal administration of omarigliptin (5 mg/Kg) showing concentration of 802.35 ng/mL ± 76.85 expressed as mean ± S.E.M.

All the validation parameters were satisfying according to US-FDA guidelines^44^. Linearity range of (50–2800 ng/mL) was enough for successful bioassay of the mentioned drugs in rats’ plasma & brain tissue. Accuracy & precision of the method was confirmed by RE and % RSD values (Table 1). Selectivity of the method was confirmed by absence of interference after comparison between blank samples (Fig. 3), zero samples (Fig. 4), LLOQ samples (Fig. 4) & *in vivo* samples (Figs 5, 6 and 7). No carry over was observed when injecting high concentration sample after the blank. The other validation results in (Table 1) confirmed that no significant matrix effect was observed, acceptable recoveries were obtained & good results were found regarding all the stability studies (R% below 15% for all variables).

OG & TG concentrations in rats’ plasma were found to be 2688.79 & 1754.79 ng/mL after 2 h from the oral administration but only OG crossed the BBB showing concentration of 621.75 ng/g in brain tissue after considering the dilution factor of ten due to its low molecular weight & lipophilic properties suggesting its repositioning as antiparkinsonian agent. The results of BBB crossing will be of interest for researchers interested in Parkinson’s disease. Intranasal brain/plasma ratio of 0.76 showed a promising targeting effect than the oral ratio of 0.23 that may be attributed to direct crossing of the drug for the olfactory region targeting the cerebrospinal fluid in addition to BBB crossing after trans-mucosal system absorption. The simple intranasal formulation was developed using sodium lauryl sulphate surfactant (2.5%, *w/v*) to solubilize the lipophilic omarigliptin with penetration enhancing & antimicrobial properties. Intranasal administration (n = 6) showed enhanced brain/plasma ratio by 3.3 folds than the oral group accompanied with 2.6 folds increase in brain glucagon-like peptide-1 (GLP-1) concentration than the control group. Parkinson’s disease (PD) is the second most common neurodegenerative disease. This investigation supported the repositioning of a once-weekly anti-diabetic safe drug for PD treatment enhancing the patient compliance as well as its economic impact as one dose per week instead of the marketed daily drugs. The ultimate objective of OG repositioning, in the underlying project, is to overcome the escalating costs, stagnant productivity and protracted timelines to bring therapeutic drugs to the PD market. Not only omarigliptin enhances the intestinal Glucagon like peptide-1 (GLP-1) but also it crosses BBB enhancing them in the brain with expected high neuroprotective effects. It is not mandatory for the gliptin to cross the BBB to enhance a neuroprotective effect as it increases GLP-1 & consequently, GLP-1 can cross the BBB. However, crossing the BBB is a promising breakthrough for gliptins^9^ that will ensure a double effect, the first from intestinal GLP-1 and the second from the brain GLP-1 especially through the intranasal route of administration that showed 3.3 folds the brain/plasma ratio. OG is the first gliptin that crossed BBB either from the oral route or from the intranasal route and it increased the brain concentration of GLP-1 significantly, which is the main finding of the present work.

Moreover, the developed method in rats’ plasma was extended to human plasma but change in species necessitated a partial validation study based on QC samples with limitation that it covered only the Cmax value and lower recoveries by around 13% were obtained because of the more complex matrix. Samples from twelve healthy volunteers were collected at 1.5 h after single oral dose of one Marizev^®^ tablet nominally containing 25 mg of OG or one Zafatek^®^ tablet nominally containing 100 mg of TG. The ethics committee of the British University in Egypt approved the experimental protocols and informed consents. Moreover, the protocol was registered in clinicaltrials.gov (ID: NCT03362398). The blood glucose levels were monitored for all the human subjects and no fluctuation was found confirming the anti-hyperglycemic effect of the drugs only in case of high glucose levels as an advantage instead of the direct hypoglycemic effect of some marketed anti-diabetics. Concentration of OG & TG in human plasma (after 1.5 h) were found to be 276.4 & 193.44 ng/mL, respectively which is in agreement with previously developed OG & TG pharmacokinetic studies^35,40,42,70–72^.

Literature review showed many cases reporting the anti-cancer effect of gliptins confirming its poly-pharmacology in addition to their anti-diabetic, anti-oxidative, anti-inflammatory & neuro-protective effects. It is reported that DPP-4 inhibition enhanced the antitumor response to melanoma and diminished tumor growth^75,76^. Sitagliptin showed reduction in breast cancer risk in women with type-2 diabetes^77^. It is documented that GLP-1 arrests cell proliferation of colon cancer cells suggesting its protective role in colon cancer^78^. Sitagliptin also reduced colon carcinogenesis in rats^79^. Vildagliptin inhibited lung tumor genesis in one study^80^. Screening results of sitagliptin and vildagliptin on colon cancer cell lines (HT-29) showed IC 50 values of 31.2 and 125 µg/mL, respectively^81^. And as *in vitro* screening for preliminary drug repositioning is preferable than *in vivo* methods^82,83^, In the present investigation, OG & TG were tested against MCF-7 breast cancer cell lines and showed IC 50 values of 125 & 250 µg/mL, respectively (Vacsera, Giza, Egypt). However, the relatively high value of IC 50 and the absence of potent anticancer activity at lower concentrations, after NCI screening (MD, USA), excluded their repositioning as potent anticancer agents.

**Figure 8:**
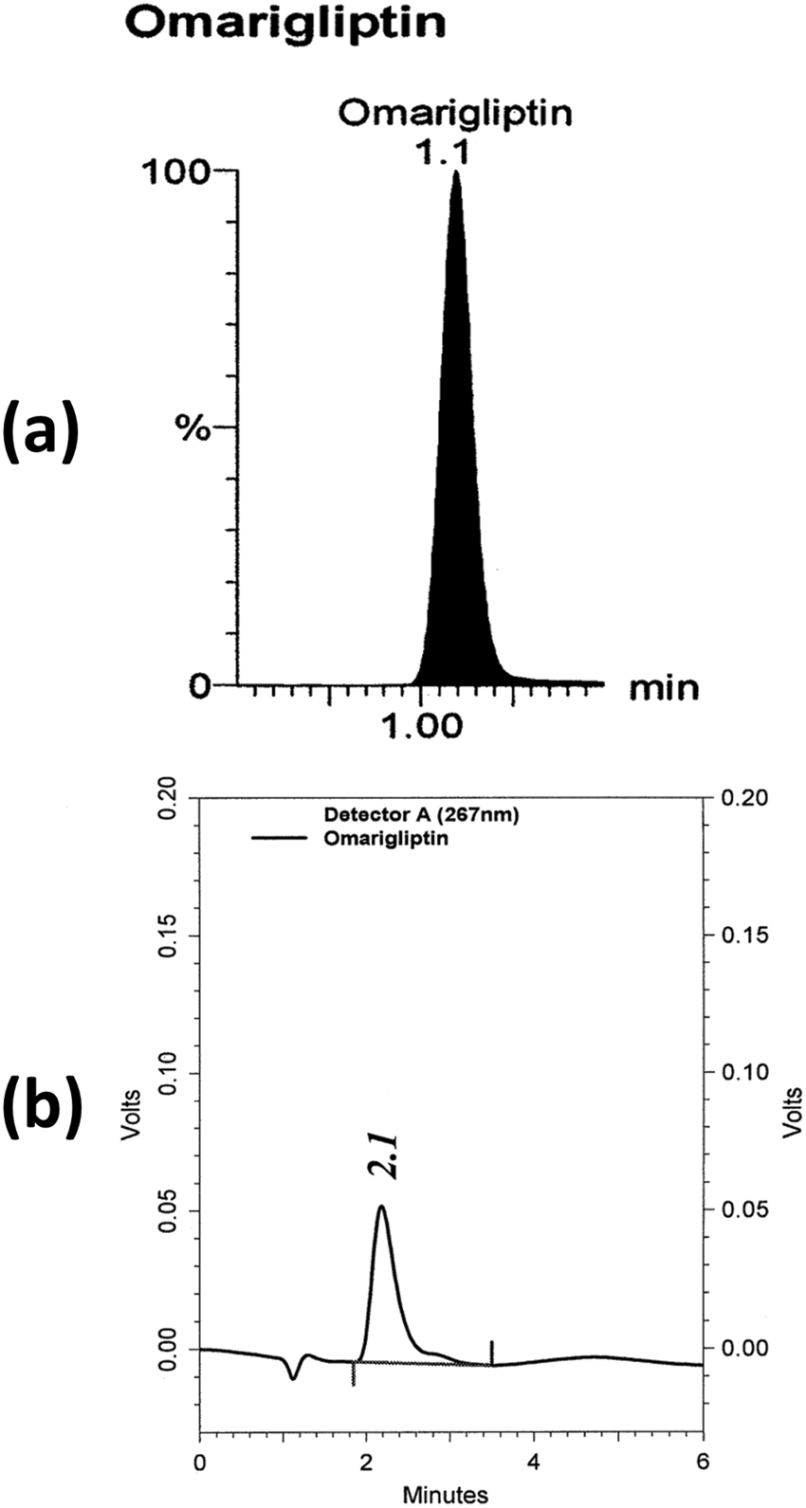
(**a**) UPLC-ESI-MS/MS chromatogram of Marizev^®^ tablet extract containing 2 µg/mL of omarigliptin at 1.1 min. (**b**) HPLC-UV chromatogram of 10 µg/mL omarigliptin in bulk at 2.1 min.

The authors previously developed LC-MS/MS & LC-UV methods for TG assay in tablets^61^ while there are no reported methods for OG assay in tablets. A simple LC-MS/MS method was developed and compared to LC-UV method at 267 nm for OG assay in pharmaceutical dosage form. All the described chromatographic conditions and mass detector parameters above were adopted for OG assay in bulk and the results are displayed in (Table 2). For the LC-UV method, C_18_ Column (4.6 × 250 mm, 5 µm) was used. The LC-MS/MS method showed higher sensitivity than LC-UV method so accuracy, precision & pharmaceutical dosage form analysis were applied successfully (Table 2). Figure 8 shows OG retention times of 1.1 & 2.1 min for the LC-MS/MS & LC-UV, respectively.

**Table 2:** Results obtained for the described UPLC-MS/MS & HPLC-UV methods for determination of OG in bulk and tablets.

Item	UPLC-MS/MS	HPLC-UV
Regression Eq.	Y = 14.561 × +310.35	Y = 1070.3 × +30.4
Correlation Co.	0.9999	0.9997
*LOD	6.67 ng/mL	0.67 µg/mL
*LOQ	20 ng/mL	2 µg/mL
Range	20–2700 ng/mL	2–10 µg/mL
Accuracy (*R%)	99.88% ± 0.7	—
Precision (^*^% RSD)	0.71%	—
Tablets (Marizev^®^)	Recovery = 98.08%	—

*Where LOD is the limit of detection, LOQ is the limit of quantification, R% is the mean recovery percent and % RSD is the percent relative standard deviation.

**Figure 9:**
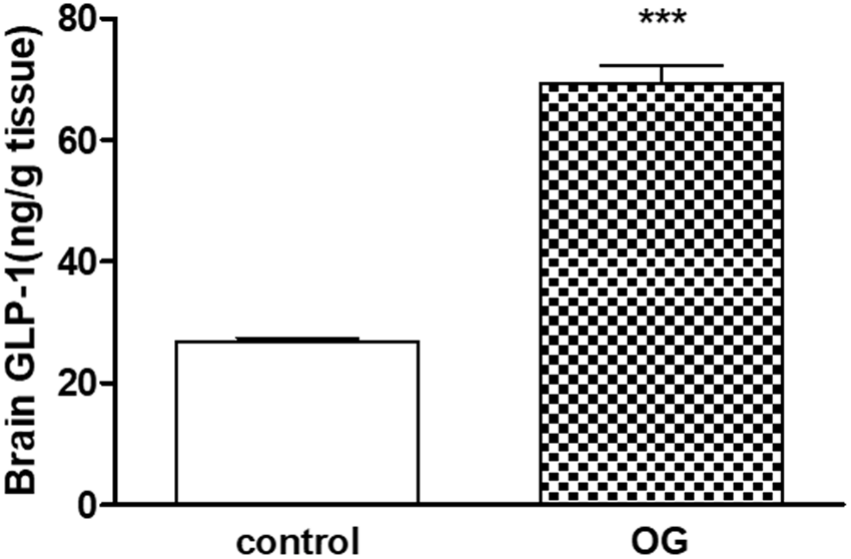
Effect of a single intranasal administration of OG (5 mg/kg) on brain GLP-1 level in rats. Data are presented as means ± S.E.M. (n = 6). ***p < 0.001 compared to control group (Student’s t-test).

Finally, Statistical analysis was performed for the effect of a single intranasal administration of OG (5 mg/kg) on brain GLP-1 level in rats. Data are presented as means ± S.E.M in Fig. 9, (n = 6). ***p < 0.001 compared to control group. Statistical analysis was performed using a software program (GraphPad Prism, version 5.01, Inc., 2007, San Diego, CA, USA). GLP-1 results were analyzed using two-tailed Student’s t-test test. Probability values of less than 0.05 were considered statistically significant.

## Conclusion

OG crossed the BBB successfully either after oral administration or intra-nasal route, which suggest its repositioning as antiparkinsonian agent due to many reasons. The first reason is that the first developed gliptins showed a reported antiparkinsonian activity. Furthermore, its mechanism of action involves rising of GLP-1 and other hormone levels by inhibiting the degrading enzyme DPP-4 & the increased GLP-1 had a reported and well established potential antiparkinsonian effect. GLP-1 is a potential candidate in modifying neurodegenerative diseases as a promising antiparkinsonian effect of DPP-4 inhibitors. Finally, as a once-weekly medication, patient compliance will be enhanced with many advantages over the daily marketed drugs especially with intranasal administration that showed enhanced brain/plasma ratio by 3.3 folds than the oral group accompanied with 2.6 folds increase in brain glucagon-like peptide-1 (GLP-1) concentration than the control group.
